# Royal Jelly and Aliskiren mutually annul their protective effects against gentamicin-induced nephrotoxicity in rats

**DOI:** 10.14202/vetworld.2020.2658-2662

**Published:** 2020-12-14

**Authors:** Mohd Alaraj

**Affiliations:** Department of Pharmacy, Faculty of Pharmacy, Middle East University, Amman, Jordan

**Keywords:** aliskiren, gentamicin, nephrotoxicity, royal jelly

## Abstract

**Background and Aim:**

Gentamicin (GM) is one of the most effective antibiotics for severe, life-threatening Gram-negative infections. Nevertheless, its clinical use has been restrained because of its nephrotoxic potential. Royal jelly (RJ) and aliskiren (ALK) can individually prevent such toxic effects. The aim of this study was to explore the protective effects of a combination treatment of RJ and ALK on GM-mediated nephrotoxicity.

**Materials and Methods:**

Thirty-two adult female. Wistar rats were divided equally into four groups: (I) Receiving normal saline; (II) GM (100 mg/kg, intraperitoneal [i.p.] injection); GM (100 mg/kg, i.p. injection) plus ALK (50 mg/kg, i.p. injection); and (IV) GM (100 mg/kg, i.p. injection) plus ALK (50 mg/kg, i.p. injection) in combination with RJ (150 mg/kg, orally). All treatments were administered daily for 10 days. The blood levels of creatinine, urea, uric acid, albumin, and total protein were measured. Then, the animals were sacrificed, and the kidneys were taken for histopathology.

**Results:**

Compared to normal control rats, GM-injected rats showed significantly (p<0.001) higher serum concentrations of uric acid, urea, and creatinine as well as evidently (p<0.001) lower blood levels of albumin and total protein. Moreover, GM administration was associated with significant renal histopathological changes. All these alterations were considerably (p<0.05) improved in GM-injected rats receiving ALK compared to rats receiving GM alone. However, when RJ was given in combination with ALK to GM-injected rats, it lessened the beneficial nephroprotective effects of both agents.

**Conclusion:**

The combination treatment of RJ and ALK is not desirable for GM-induced nephrotoxicity. Further studies are crucial to accurately explore the precise mechanism of RJ antagonistic interaction with ALK.

## Introduction

Gentamicin (GM), an aminoglycoside antibiotic, is utilized for the management of severe, life-threatening Gram-negative infections. Nevertheless, the ideal clinical usage of GM has been diminished by a number of side effects, the most significant of which are ototoxicity [1], hepatotoxicity [2], and nephrotoxicity [3]. GM induces nephrotoxicity in a dose-dependent manner in 30% of patients treated for more than 7 days, and this toxicity manifests as proteinuria, hypoalbuminemia, or severe proximal renal tubular necrosis, leading to declining in renal function and/or renal failure [4].

Although the precise mechanism of GM-induced nephrotoxicity is still unknown, it is supposed that the toxicity can be intermediated by the generation of a reactive oxygen species (ROS), protein oxidation, lipid peroxidation [5], and excessive activity of a renin-angiotensin system (RAS) [6]. Indeed, RAS has been demonstrated to have a vital function in the pathogenesis of numerous cardiovascular and renal disorders [7]. A few studies have revealed that angiotensin-converting enzyme inhibitors (ACEIs) [8], angiotensin receptor blockers (ARBs) [9], and renin inhibitors might exert a protective effect against GM-induced nephrotoxicity [10]. However, Aliskiren (ALK) – a renin inhibitor – demonstrated superior protection against doxorubicin nephrotoxicity compared to other RAS blockers [11]. This may be due to the characteristic properties of ALK. Specifically, ALK does not have an angiotensin-converting enzyme (ACE) escape action; instead, it stops the formation of both angiotensin I and angiotensin II and produces an additional blockade of RAS without a compensatory rise in the plasma renin activity [12].

Royal jelly (RJ), a milky natural substance secreted by bees, has been shown to reduce the drug-induced nephrotoxicity effect of many drugs, including cisplatin [13], cadmium [14], and GM [15,16]. This substance contains fatty acids (such as 10-hydroxy-2-decenoic acid, also known as 10-HDA); proteins (such as royalactin); monosaccharides; lipids; free amino acids; minerals; and some vitamins [14]. The beneficial effects of RJ might be attributed to its high levels of antioxidants, free radical scavenging capacity, anti-inflammatory properties, and other beneficial effects [14].

However, ALK failed to reach complete renoprotection; therefore, this work aimed to investigate the effects of combining the beneficial properties of RJ with the protective activity of ALK against GM-induced nephrotoxicity.

## Materials and Methods

### Ethical approval

The protocol of this study was approved by the Ethics Board of Animal Experiments, University of Hail. All experimental procedures were conducted according to the guidelines set by the World Health Organization (Geneva, Switzerland).

### Products and chemicals

Egyptian RJ was purchased from the Wadi Al Nahil for Honey and Oud, Hail, Saudi Arabia, and was stored at −20°C until use. GM sulfate was provided by Abbott India Ltd., Mumbai, India. ALK was obtained from Novartis International AG., Basel, Switzerland. All of the other chemicals used were of analytical grade.

### Animals

The experiments were carried out on 32 female Wistar albino rats (150-200 g) (10-12 weeks old). Healthy rats were obtained from King Saud University, Riyadh, Saudi Arabia. The rats were accommodated in colony cages with access to standard commercial food and tap water. The experiments started after 7-day acclimatization to standardized laboratory conditions (22-24°C and a 12 h light/dark cycle).

### Grouping and experimental induction/treatment procedures

Animals were randomized into four groups (n=8) and treated for 10 days, as follows: I. Control group: The animals were administered intraperitoneally (i.p.) with 0.09 normal saline; II. GM group: The rats were treated (i.p.) with GM at a dose of 100 mg/kg/day [15]; III. GM+ALK group: The rats were injected (i.p.) with ALK (dissolved in normal saline at a dose of 50 mg/kg/day) for 30 min before the injection of GM [11]; and IV. GM+ALK+RJ group: The rats were administered orally with RJ (dissolved in normal saline at a dose of 150 mg/kg/day) [17] together with i.p. ALK (50 mg/kg/day) 30 min before GM injection.

### Biochemical estimation in plasma

All animals were sacrificed after 24 h from the last dose. Blood samples were collected directly from the heart, and the sera samples were separated to test the biochemical indices. Assessment of renal function was carried out using the following indices: Blood urea nitrogen (BUN) level, serum creatinine (SCr) level, total protein, uric acid, and albumin. All indices were estimated by enzymatic kits using an ultraviolet (UV)–visible spectrophotometer (Shimadzu UV-1601, 84, Japan) according to the instruction of the manufacturer (Human Diagnostics, Germany).

### Histopathological examination

Following scarification, the kidneys were immediately removed, dissected, and washed with a Phosphate-buffered saline (pH 7.4) to remove the blood stains and clots completely. Afterward, the samples were fixed in 10% formalin and embedded in molten paraffin wax followed by cutting into sections with a microtome. Deparaffinized pieces were then stained with hematoxylin and eosin to investigate microscopically for histopathological alterations.

### Statistical analysis

Statistical analyses were performed using SPSS version 20 (Chicago, IL, USA). Data were expressed as the mean values±SEM. The Kolmogorov–Smirnov test was used to measure the normality of the numeric variables. Differences among the groups were obtained using one-way analysis of variance (ANOVA) tests with Tukey-Kramer as the *post hoc* test. p<0.05 was considered to be the minimal level of significance.

## Results

No losses or extraordinary marks of external toxicity were detected in the groups of rats administered GM, either alone or in combination with other agents.

### Biochemical analysis (Table-1)

**Table-1:** Effect of ALK and “RJ+ALK” on kidney linked plasma biomarkers of GM intoxicated female rats.

Group	Uric acid (mg/dL)	Urea (mg/dL)	Creatinine (mg/dL)	Albumin (mg/dL)	Total protein (mg/dL)
Control	2.94±0.05^a^	38.00±4.00^a^	0.47±0.005^a^	4.05±0.163^a^	6.55±0.22^a^
GM	5.79±0.08^c^	142.00±8.14^c^	0.89±0.455^b^	2.60±0.159^c^	4.19±0.19^c^
GM+ALK	4.35±0.17^b^	54.13±6.13^b^	0.52±0.373^a^	3.23±0.167^b^	5.16±0.20^b^
GM+ALK+RJ	5.17±0.147^c^	124.13±5.4^c^	0.81±0.51^b^	2.92±0.161^bc^	4.38±0.189^c^

GM=Gentamicin (100 mg/kg, i.p.), ALK=Aliskiren (50 mg/kg, i.p.), RJ=Royal jelly (150 mg/kg orally). Data were expressed as means±SEM for eight rats in each group. The different letters in the same column indicate statistically significant different means according to Tukey-Kramer test

One-way ANOVA revealed a highly significant difference in the serum level of the studied parameters in the different animal groups (p<0.001). For serum uric acid, BUN, and SCr, a highly significant increase was detected in the GM-treated group compared with those of the control. For the serum albumin and total proteins, a significantly lower level was detected in the GM group compared with those of the control animals treated with 0.09 normal saline.

Rats administered ALK (50 mg/kg b.w./day for 10 days) and GM demonstrated a significant decrease (p<0.05) in the SCr, BUN, and uric acid levels when compared to those of the GM group. The albumin and total protein blood levels of the ALK plus GM group were significantly lower than those of the GM-treated rats (p<0.05).

Contrariwise, the combined treatment of ALK with RJ (GM+ALK+RJ group) at the above-mentioned doses caused statistically insignificant restoration of GM alterations in the serum levels of SCr, BUN, uric acid, albumin, and total protein, in comparison with the GM-treated group animals.

### Histopathological results

**Figure-1:**
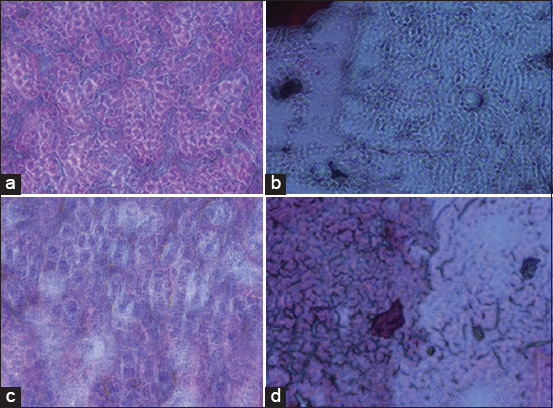
(a) Normal tubules and glomerulus in kidney cortex hematoxylin and eosin (H&E) 200× (control group). (b) Severe tubular necrosis, tubular degeneration, and epithelial vacuolization in the proximal tubules H&E 200× (GM-treated group). (c) Slight tubular necrosis H&E 200× (GM+Aliskiren [ALK] treated group). (d) Massive necrosis with minor prevention of gentamicin alterations H&E 200× (GM+Royal jelly+ALK).

Histopathologic analysis exhibited that there were no abnormal morphological changes in the kidney specimens of the control group (Figure-1a). The kidney samples from rats receiving GM (Figure-1b) showed substantial degeneration of the tubular epithelium with the occurrence of an eosinophilic hyaline cast in the lumen with dilatation of the renal tubules. Thickening of the basement membrane of the glomeruli and congestion of the glomerular tuft also occurred, sometimes with detachment and disappearance of the tubular epithelium of the glomerular membrane. In addition, vacuolization of the glomerular epithelium was observed.

Nevertheless, administration of ALK with GM injection markedly attenuated renal injury but maintained slight tubular necrosis (Figure-1c). However, as shown in Figure-1d, rats coadministered RJ with ALK showed massive necrosis with minor prevention of GM morphological changes.

## Discussion

Renal failure refers to the state where the kidneys cannot excrete nitrogenous waste products; that is, BUN and SCr [18]. The levels of SCr and BUN depend on the glomerular filtration rate (GFR). Renal failure decreases the GFR of SCr and BUN, and consequently, the blood concentrations of these indices rise. Nephrotoxicity is initiated if the kidneys are repeatedly exposed to drug-induced oxidative stress and inflammation [19]. For this condition, interference by an antioxidant and anti-inflammatory agent is vital. Therefore, the current study evaluated the potentials of ALK alone and the ALK/RJ combination to ameliorate nephrotoxicity. Thus, the present study was carried out by inducing nephrotoxicity in rats utilizing GM, which is one of the most widely used experimental models by which to examine the nephroprotective effect of natural products and drugs [20].

Nephrotoxicity was induced in this study by GM injections at a dose of 100 mg/kg for 10 successive days, and it was documented through significant elevations of the levels of SCr, uric acid, and BUN in GM-treated animals, as was previously reported [21]. Similar to other reports [22,23], GM also caused a significant decrease of the blood levels of total protein and serum albumin, which is probably attributed to an extraordinary escape resulting from the hypercellularity of both the glomeruli and tubules.

Consistent with the previous investigations [21], the current study also demonstrated marked histological changes in kidney tissues characterized by severe degenerative changes in the renal glomeruli and necrosis of the proximal convoluted tubule. Although the pathogenesis of GM-induced renal dysfunction is not completely understood, it has been proposed that GM causes increased lipid peroxidation properties and triggers the inhibition of antioxidant enzyme activities in kidney tissues. MDA concentrations were found to be elevated in the kidney tissues of GM-treated rats [15,21]. Contrarily, the glutathione (GSH) concentrations reduced in the kidney tissues of GM-treated rats [21].

Overstimulation of the renin-angiotensin (renin-angiotensin-aldosterone system [RAAS]) system induced by GM may be involved in GM-triggered apoptosis. Therefore, blocking this system has been reported to protect GM nephropathy [10]. Indeed, this study revealed that administration of ALK alone to GM-treated animals induced a significant decrease in the SCR, uric acid, and BUN concentrations, which might be attributed to the restoration of glomerular filtration impairment caused by GM. The present study also demonstrated that hypoalbuminemia, which is a characteristic feature of GM-induced nephrotoxicity, was improved by ALK. Moreover, ALK significantly diminished the histopathological changes. Therefore, it was proposed that ALK alone recovers renal function by countering GM-induced nephropathy.

Blocking of the RAAS system by various drugs has been found to protect against nephrotoxicity in various animal models, as well as in human diabetic nephropathy. The nephroprotective activity of these agents is probably caused by the inhibition of ROS formation induced by RAAS activation. Indeed, ALK has revealed a renoprotective effect against various animal model drugs (such as gentamicin and adenine) [10,24]. The results obtained by Bae *et al*. [10], which are in agreement with the findings of this study, suggest that the protective activity of ALK against GM nephrotoxicity is attributed to the repression of apoptosis and inflammation through prevention of the expression of proapoptotic markers and a reduction of elevated levels of inflammatory cytokines and adhesion molecules, which were increased in GM-treated kidneys. However, a study by Lopez-Novoa *et al*. [25] showed that decreasing glomerular filtration mediated by mesangial cells likely activates RAAS and the formation of angiotensin II, which then triggers the formation of ROS, especially superoxide in the renal tubule and smooth muscle cells. Taken together, these and other studies suggest that the renoprotective activity of ALK probably occurred through exerting anti-inflammatory and antioxidant activities.

RJ is an extremely effective exogenous antioxidant and, [14] thus, is capable of scavenging free radicals such as superoxide radicals, hydroxyl radicals, and 1,1-diphenyl-2-picrylhydrazyl hydrate radicals. This substance also has been shown to decrease malondialdehyde (MDA) concentrations and to upregulate the levels of reduced GSH [14]. Moreover, clinical trials have demonstrated that RJ decreased oxidative stress through improved concentrations of MDA and activities of GSH peroxidase and superoxide dismutase in the erythrocytes of diabetic patients [26]. In parallel, RJ was found to have an anti-inflammatory effect in addition to its antioxidant effect. Several studies have shown that pre-treatment with RJ reduces tumor necrosis factor-α, interleukin (IL)-1, and IL-6 without inducing the cytotoxic properties of the macrophages [27].

The previous studies proved that RJ can mitigate nephrotoxicity induced by GM in numerous experimental models, in which it also normalized the serum urea, creatinine, and histological changes, as well as prevented oxidative stress [14-16].

However, synergistic or additive ALK combined with other antioxidants or other RAS blockers has been reported in animal models [28,29]; therefore, estimating the kidney restoration capability of the ALK/RJ combination was considered to be the main aim of the present work and is the first report in this area. Nevertheless, the results of the current study demonstrate that combined RJ/ALK did not attenuate GM-induced nephrotoxicity compared with ALK monotherapy in this rat model and instead indicates an antagonistic renoprotective effect.

An earlier study revealed that RJ contains an ACE [30] and renin [31] inhibitory peptides. Therefore, one possible explanation for the obtained results of this study is that adding RJ to ALK inhibits RAAS more efficiently, thus leading to a marked decreased in the renal perfusion pressure, consequential failure of the glomerular filtration, and aggravated GM-induced nephrotoxicity. Indeed, earlier reports demonstrated that a dual RAAS blockade had been associated with an increased incidence of hyperkalemia, hypotension, and renal dysfunction [32].

It is also possible that RJ stimulates the prorenin receptor, thus activating cyclooxygenase-2 and the fibrotic pathways, which consequently results in proteinuria, glomerulosclerosis, and nephropathy [33]. These adverse outcomes could counterbalance any favorable outcome of the combined treatment.

RJ is a health food that is widely used as a supplement for managing many diseases, including neurodegenerative disorders, cancer, diabetes, and atherosclerosis [34]. Therefore, further studies are essential to determining whether RJ counteracts the beneficial effects of other RAAS blockers, such as ACEI and ARBs, in terms of their renoprotective and other beneficial properties, particularly since these drugs are routinely used for diabetic nephropathy [35].

## Conclusion

Considering these results, the addition of RJ to a RAAS inhibitor in GM-treated subjects should not be encouraged. Further studies are needed to accurately explore the precise mechanism of RJ interaction with RAAS blockers.

## Author’s Contributions

MA designed the study, drafted the manuscript, performed all the experimental procedures, and conducted data analysis and interpretation. The author read and approved the final version of the manuscript.
